# Mutation spectrum of the *FZD-4*, *TSPAN12* AND *ZNF408* genes in Indian FEVR patients

**DOI:** 10.1186/s12886-016-0236-y

**Published:** 2016-06-17

**Authors:** Ganeswara Rao Musada, Hameed Syed, Subhadra Jalali, Subhabrata Chakrabarti, Inderjeet Kaur

**Affiliations:** Kallam Anji Reddy Molecular Genetics Laboratory, Brien Holden Eye Research Centre, L V Prasad Eye Institute (KAR Campus), Road#2, Banjara Hills, Hyderabad, 500034 India; Smt. Kanuri Santhamma Centre for Vitreo Retinal Diseases, LV Prasad Eye Institute, Hyderabad, India

**Keywords:** Retina, Abnormal angiogenesis, Mutation screening, Candidate genes

## Abstract

**Background:**

Mutations in candidate genes that encode for a ligand (*NDP*) and receptor complex (*FZD4, LRP5 and TSPAN12*) in the Norrin β-catenin signaling pathway are involved in the pathogenesis of familial exudative vitreoretinopathy (FEVR, MIM # 133780). Recently, a transcription factor (*ZNF408*) has also been implicated in FEVR. We had earlier characterized the variations in *NDP* among FEVR patients from India. The present study aimed at understanding the involvement of the remaining genes (*FZD4*, *TSPAN12* and *ZNF408*) in the same cohort.

**Methods:**

The DNA of 110 unrelated FEVR patients and 115 unaffected controls were screened for variations in the entire coding and untranslated regions of these 3 genes by resequencing. Segregation of the disease-associated variants was assessed in the family members of the probands. The effect of the observed missense changes were further analyzed by SIFT and PolyPhen-2 scores.

**Results:**

The screening of *FZD4, TSPAN12* and *ZNF408* genes identified 11 different mutations in 15/110 FEVR probands. Of the 11 identified mutations, 6 mutations were novel. The detected missense mutations were mainly located in the domains which are functionally crucial for the formation of ligand-receptor complex and as they replaced evolutionarily highly conserved amino acids with a SIFT score < 0.005, they are predicted to be pathogenic. Additionally 2 novel and 16 reported single nucleotide polymorphisms (SNP) were also detected.

**Conclusions:**

Our genetic screening revealed varying mutation frequencies in the *FZD4* (8.0 %)*, TSPAN12* (5.4 %) and *ZNF408* (2.7 %) genes among the FEVR patients, indicating their potential role in the disease pathogenesis. The observed mutations segregated with the disease phenotype and exhibited variable expressivity. The mutations in *FZD4* and *TSPAN12* were involved in autosomal dominant and autosomal recessive families and further validates the involvement of these gene in FEVR development.

**Electronic supplementary material:**

The online version of this article (doi:10.1186/s12886-016-0236-y) contains supplementary material, which is available to authorized users.

## Background

FEVR is a potentially blinding eye disorder caused by mutations in genes that play a crucial role in the development of the normal retinal vasculature [1–10]. It is primarily characterized by a failed development of the peripheral retinal vasculature on the temporal side of the retina [11–13]. This feature is asymptomatic and observed in most patients without any further progression during their life time [7, 11, 12]. However, it might lead to secondary clinical complications including neovascularization, retinal traction with ectopic macula, exudation, subtotal and total retinal detachment in some patients [7, 11, 12]. The age of onset in FEVR is variable but the severe disease phenotype is usually observed in the first or second decade of a patient's life. A highly variable expressivity between the two eyes of the same patient and among the different family members is another commonly reported feature of this disease [7, 11, 12].

So far, mutations in 6 candidate genes have been attributed to the development of FEVR including Norrie disease pseudoglioma (*NDP;* OMIM 300658) [1, 9, 10, 14, 15], Frizzled-4 (*FZD4;* OMIM 604579) [1, 2, 7, 8, 14–20], Low density lipoprotein receptor like protein 5 (*LRP5;* OMIM 603576) [1, 2, 6, 14–16, 19, 21], Tetraspanin-12 (*TSPAN12;* OMIM 613138) [1, 2, 4, 5, 22–25], Zinc finger prortein-408 (*ZNF408*) [1, 3] and Kinesin family member 11 (*KIF11*) [26, 27]. Mutations in these genes have resulted in autosomal dominant (*FZD4, LRP5, TSPAN12, ZNF408* and *KIF11*)[[3, 7, 8, 14–16, 24, 26, 27], autosomal recessive (*LRP5, TSPAN12*) [21, 23] and X-linked (*NDP*) FEVR [9, 10] and have accounted for ~50 % of all patients [1–3]. Among these, the autosomal dominant mode of inheritance has been widely reported. The proteins encoded by FEVR candidate genes (excluding *ZNF408* and *KIF11*) have been shown to be involved in the formation of a signal transduction complex of Norrin-β catenin signaling pathway [28–32]. The knockout mouse models of these genes have exhibited phenotype resembling FEVR and have provided further evidence on the role of this signaling pathway in the formation, maturation and maintenance of intraretinal vasculature [28–31, 33–37]. Norrin, a protein encoded by *NDP* gene being similar to canonical-Wnt signaling pathway, acts as a ligand and interacts with FZD4-LRP5 receptor complex. This interaction further stabilizes the cytoplasmic β catenin molecules to induce the T-cell factor/lymphoid enhancer factor (TCF/LEF) mediated gene expression after its translocation into the nucleus [30–32]. TSPAN12 enhances the Norrin signaling by augmentation of FZD4 multimerization [28]. However, the roles of the *ZNF408* and *KIF11* genes in the developmental retinal angiogenesis and Norrin- β catenin signaling pathway is yet unknown.

The FZD4 is a 537 amino acid protein containing an N-terminal extracellular domain (composed of signal sequence, FZ domain and a linker region), 7 transmembrane hydrophobic helical domains, 3 extracellular, 3 intracellular loops and a C terminal cytoplasmic domain [38, 39]. The extracellular FZ domain is cysteine rich (CRD) and is essential for the recognition and binding to FZD4 specific ligands [40, 41]. The cytoplasmic C terminal region contains two highly conserved PDZ binding domains (postsynaptic density 95/disc-large/zona occludens-1: KTXXXW and ETVV) and play a crucial role in recruiting downstream regulator proteins to induce the signaling mechanism [39]. The *TSPAN12* gene is located on chromosome 7q31 and encodes for a 305 amino acid transmembrane protein. Structurally, this protein contains four transmembrane domains connected by two extra cellular loops (ECL-1 and 2) and an intra cellular loop, and intracellular N and C terminals. The ECL-1 is small compared to the ECL-2 [4, 5, 22].

Recently, Collin et al. [3] functionally characterized this new candidate gene *ZNF408* in FEVR. A novel founder mutation was observed in two Dutch families and further screening of 132 patients revealed another mutation in a Japanese patient. The *ZNF408* gene encodes for a 720 amino acid protein and predicted to contain ten zinc finger DNA binding domains. Based on the presence of zinc finger domains, this protein was suggested to be a transcription factor [3]. Functional analysis of mutant proteins indicated a dominant negative disease mechanism and subsequent knockdown of *Znf408* in zebrafish, suggested its putative role in retinal vasculogenesis.

So far, 88 different mutations have been reported in *FZD4* (*n* = 58)*, TSPAN12* (*n* = 27) and *ZNF408* (*n* = 3) in patients with FEVR and other vitreo-retinal abnormalities. These genes have been quite well characterized in Caucasian, Chinese and Japanese FEVR patients. However, in the Indian context, there is only a single report from a relatively smaller cohort of FEVR patients on *FZD4* gene screening [42] (Nallathambi J et al.,). Thus, the present study was undertaken to explore the involvement of the *FZD4*, *TSPAN12* and *ZNF408* genes and to assess their mutation spectrum in a large cohort of Indian FEVR patients.

## Methods

The study was approved by the Institutional Review Board (IRB) of L.V. Prasad Eye Institute, Hyderabad, India, (Ref no LEC06104) and adhered to tenets of the Declaration of Helsinki. We recruited 110 unrelated FEVR cases (34 familial and 76 sporadic) along with their family members after obtaining a written informed consent. All the patients had a history of full term birth. A senior ophthalmologist examined all the patients and diagnosed the disease based on indirect ophthalmoscopic examination, B-scan ultrasonography and fundus fluorescein angiography in selective patients. As described previously, the diagnosis of FEVR was confirmed based on the presence of bilateral peripheral retinal avascularity along with or without any of the ocular features such as subretinal exudation, neovascularization, vitreoretinal traction, retinal folds, ectopic macula and partial or total retinal detachments [7, 12, 20]. The different stages of the disease were ascertained based on the Pendergast and Trese classification [12]. The diagnosis and staging of the disease was independently confirmed by another ophthalmologist through evaluation of the medical records and fundus photographs of the patients. Additionally, 115 ethnically matched normal subjects aged over 60 years and without any present and past history of retinal disorders nor signs or symptoms of FEVR or any other ocular or systemic conditions were enrolled as controls. They were generally subjects with senile cataract who were also evaluated for any other ocular complications through a comprehensive eye examination.

### Mutation screening of the *FZD4* and *TSPAN12* genes

Genomic DNA was extracted from peripheral blood leucocytes using standard protocols of Phenol-Chloroform extraction method [43]. Mutation screening of entire coding and intron-exon boundaries of *FZD4*, *TSPAN12* and *ZNF408* genes were accomplished by polymerase chain reaction (PCR) based amplification of these regions with 26 different sets of overlapping primers followed by resequencing on an automated DNA sequencer ABI 3130 XL (Applied Biosystems, Foster City, CA) using the Big Dye chemistry (version 3.1) following the manufacturer’s guidelines. The primers used for PCR and their optimum annealing temperatures are listed in Additional file 1: Table S1. The observed variations were further validated by resequencing. Samples of the available family members of the respective probands in the FEVR-affected families were also screened for assessing the segregation of the identified variations. In addition to the screening of the observed variants in the ethnically matched controls, their allele frequencies were also checked in the dbSNP [44], ESP5400 [45], NIEHS95 [46] and ExAC [47] databases. All the identified non-synonymous substitutions were assessed to predict their functional significance by computational prediction algorithims; SIFT [48], PolyPhen-2 [49], and Mutation Taster [50]. Multiple sequence alignments were carried out using Clustal W to determine the evolutionary conservation of wild type amino acid residues at the position of substitutions [51]. Evolutionarily non-conservative missense changes predicted as deleterious by at least two computational tools were considered as potentially pathogenic mutations. The remaining missense changes were categorized as variants of unknown significance.

## Results

**Fig. 1:**
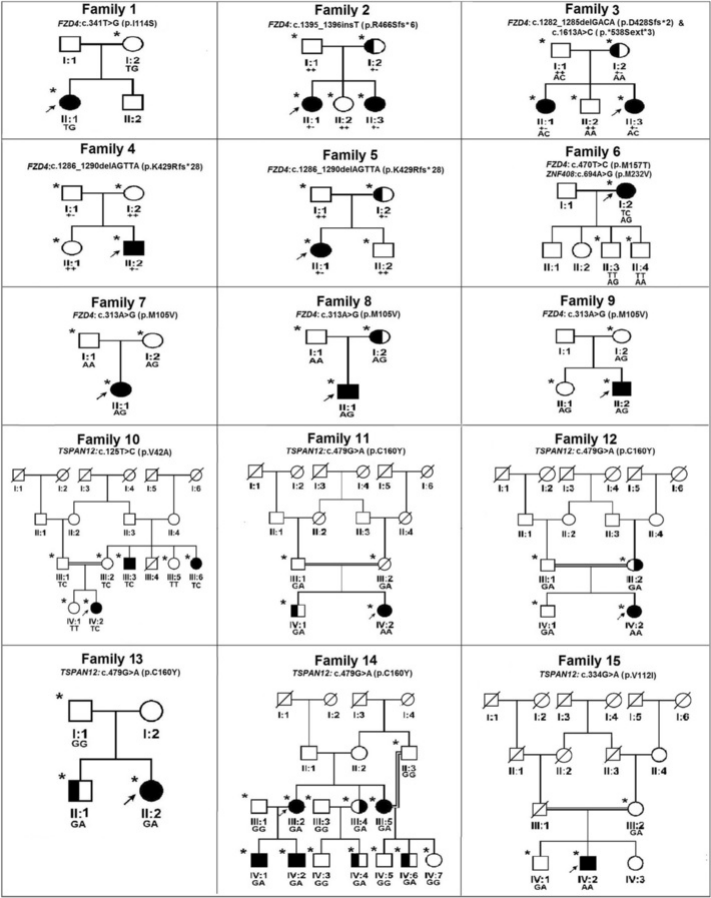
Pedigrees of the FEVR families with nucleotide changes in *FZD4* and *TSPAN12* genes. Completely shaded symbols represent severe stages of the disease. Open symbols represent unaffected individuals. Asterisk (*) over the pedigree symbols represent the individuals screened for nucleotide changes in *FZD4* and *TSPAN12* genes. - indicates the presence of deletion/insertion and + indicates the wild type allele. The identified nucleotide change is represented above each pedigree

The screening of the entire coding and flanking regions of *FZD4, TSPAN12* and *ZNF408* genes revealed a total of 11 potentially pathogenic mutations in 15 different FEVR probands (Table 1 and Fig. 1). Of these 11 mutations, six were novel. The novel mutations included a missense change (c.341 T > G; p.Ile114Ser), a single base pair insertion (c.1395_1396insT; p.Arg466Serfs*6) and a non-stop change (c.1613A > C; p.*538Serext*2) in the *FZD4* gene. Three novel mutations c.125 T > C (p.Val42Ala), c.479G > A (p.Cys160Tyr) and c.2145G > T (p. Glu715Asp) were also observed in the *TSPAN12* and *ZNF408,* respectively (Table 1 and Fig. 1). In addition to these novel changes, two previously reported missense mutations; c.313A > G; [p.Met105Val] [1, 2, 16, 18, 20] and c.470 T > C [p.Met157Thr] [2], two small base pair deletions; c.1282-1285delGACA [p.Asp428Serfs*2] [1, 2, 15, 16] and c.1286-1290delAGTTA [p.Lys429Argfs*28] [52] in the *FZD4* gene; a rare variant in *TSPAN12* gene*;* c.334G > A [p.Val112Ile] and two novel missense changes (c.130C > T [p.Pro44Ser] and c.694A > G [p.Met232Val]) in *ZNF408* of unknown significance were also observed. None of these mutations were observed in the 115 normal controls. Apart from these mutations, three reported single nucleotide polymorphisms (SNPs); c.502C > T [rs61735303], c.576C > T [rs2011686860] and c.1078 A > G [rs530613772] were observed in *FZD4* gene. Two intronic (IVS2 + 24C > G [rs545602315] and IVS6 + 19G > C) and five 3′ UTR single nucleotide substitutions (c.*39C > T [rs41622], c.*334A > T [rs545129654], c.*1010 T > G, c.*1140 T > A and c*1243A > T [rs189221112]) were observed in the *TSPAN12* gene. Likewise, two novel 5′ UTR variations (c. -214_-210delGAATC and -111C > A) and six SNPs (c.402A > G [rs561320549], c.408C > A, c.576_587del12bp [rs148055528], c.581_592del12bp [rs72461400] c.1971A > G [rs376536252] c.1850C > A [rs547169524] were observed in *ZNF408*.

**Table 1:** Mutations observed in FZD4, TSPAN12 and ZNF408 genes in FEVR patients

Family number	Gene/Exon	cDNA change^a^	Protein change^b^	In silico analysis			Occurrence in		Novel/ Reported	Functional significance
				SIFT (score)	PolyPhen-2 (score)	Mutation Taster (probability value)	Patients	Controls		
1	*FZD4/*2	c.341T>G	p.Ile114Ser	Damaging (0.00)	Probably damaging (0.996)	Disease causing (0.9999)	1/110	0/115	Novel	Pathogenic
2	*FZD4/*2	c.1395_ 1396insT	p.Arg466Ser fs*6	-	-	-	1/110	0/115	Novel	Pathogenic
3	*FZD4/*2	c.1613A>C	p.*538Serext*2	-	-	-	1/110	0/115	Novel	Pathogenic
		c.1286-1290 delAGTTA	p.Lys429Arg fs*28	-	-	-	1/110	0/115	Reported [52]	Pathogenic
4	*FZD4/*2	c.1282_1285 delGACA	p.Asp428Ser fs*2	-	-	-	2/110	0/115	Reported [1, 2, 15, 16]	Pathogenic
5										
6	*FZD4/*2	c.470T>C	p.Met157Thr	Damaging (0.039)	Benign (0.256)	Disease causing (0.9999)	1/110	0/115	Reported [2]	Pathogenic
	*ZNF408/5*	c.694A>G	p.Met232Val	Tolerated (0.508)	Benign (0.000)	Polymorphism (0.9999)	1/110	0/115	Novel	Unknown significance
7	*FZD4/2*	c.313A>G	p.Met105Val	Tolerated (0.858)	Possibly damaging (0.793)	Disease causing (0.9999)	3/110	0/115	Reported [1, 2, 16, 18, 20]	Pathogenic
8										
9										
10	*TSPAN12/*3	c.125T>C	p.Val42Ala	Damaging (0.023)	Benign (0.127)	Disease causing (0.9998)	1/110	0/115	Novel	Pathogenic
11	*TSPAN12/*7	c.479G>A	p.Cys160Tyr	Damaging (0.000)	Probably damaging (1.000)	Disease causing (0.9999)	4/110	0/115	Novel	Pathogenic
12										
13										
14										
15	*TSPAN12/*5	c.334G>A	p.Val112Ile	Tolerated (0.338)	Possibly damaging (0.533)	Disease causing (0.9999)	1/110	0/115	Reported [47]	Pathogenic
16	*ZNF408/2*	c.130C>T	p.Pro44Ser	Tolerated (0.054)	Benign (0.062)	Polymorphism (0.9999)	1/110	0/115	Novel	Unknown significance
17	*ZNF408/5*	c.2145G>T	p.Glu715Asp	Damaging (0.003)	Probably damaging (0.966)	Disease causing (0.888)	1/110	0/115	Novel	Pathogenic

^*a*^NCBI Reference Sequences for FZD4, TSPAN12 and ZNF408 mRNA are NM_012193.2, NM_012338.3 and *NM_024741.2* respectively. ^b^ NCBI Reference Sequences for FZD4, TSPAN12 and ZNF408 proteins are NM_036325.2, NP_036470.1 and NP*_079017.1* respectively. The effect of missense changes were predicted using SIFT, PolyPhen-2 and Mutation Taster online prediction tools. In SIFT, the scores less than 0.05 were considered as damaging. In PolyPhen-2, the scores less than 0.5 were considered as disease causing

All the nucleotide changes observed in *FZD4* gene were heterozygous. Excluding the c.313A > G (p.Met105Val) and c.1286-1290delAGTTA (p.Lys429Argfs*28) mutations, the remaining mutations were observed in one proband each. A missense p.Met105Val mutation was observed in three different probands and likewise a frameshift p.Lys429Argfs*28 mutation was observed in two different probands. Interestingly, a FEVR proband harbored two changes: c.1282-1285delGACA (p.Asp428Serfs*2) and c.1613A > C (p.*538Serext*2) in the *FZD4* gene.

In *TSPAN12*, two mutations c.125 T > C (p.Val42Ala) and c.334G > A (p.Val112Ile) were identified in one proband each, while the c.479G > A (p.Cys160Tyr) mutation was observed in four different probands, wherein, two of them harbored heterozygous changes and the remaining were homozygous. The p.Val112Ile mutation has also been identified in homozygous condition whereas, p.Val42Ala mutation was observed in heterozygous condition. The wild type valine residue at codon 42 was highly conserved across different species. However, this change was predicted to be non-pathogenic by PolyPhen-2 whereas, Mutation Taster and SIFT indicated for the harmful effect of this substitution.

**Fig. 2:**
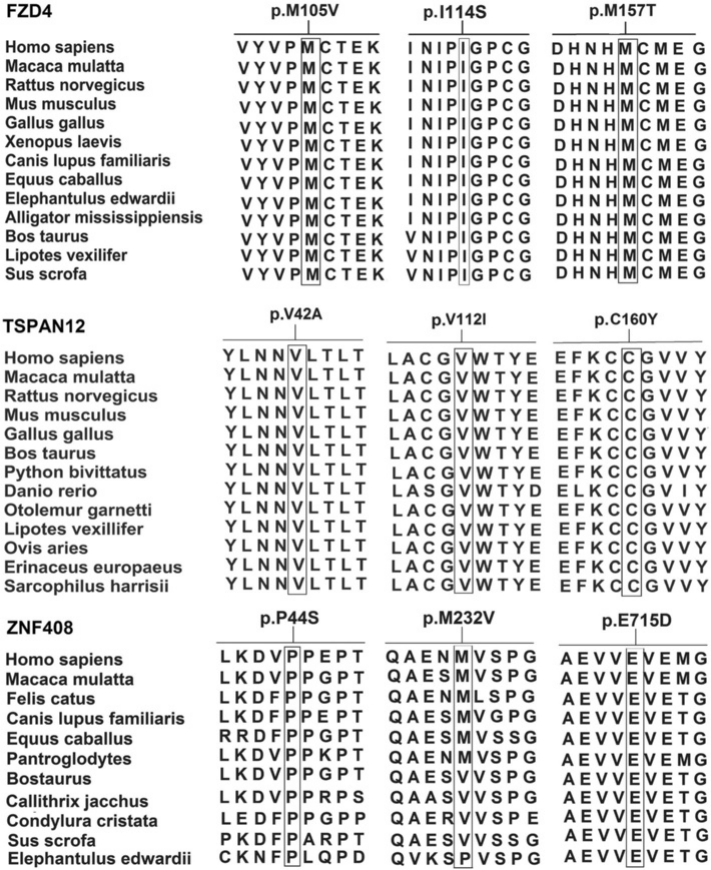
Multiple sequence alignment showing the conservation of wild type residues in respect to identified missense changes in FZD4 protein, TSPAN12, and ZNF408 across various species. Except p.M232V of ZNF408 remaining all the missense mutations were highly conserved across different species. Reference sequences of the FZD4 orthologues: Homo sapiens; AAR23924.1, Macaca mulatta; NP_001253804.1, Rattus norvegicus; NP_072145.1, Mus musculus; NP_032081.3, Gallus gallus; NP_989430.1, Xenopus laevis; NP_001083922.1, Canis lupus familiaris; XP_005633439.1, Equus caballus; XP_001489854.1, Elephantulus edwardii; XP_006895316.1, Alligator mississippiensis; XP_006261662.1, Bos taurus; NP_001193198.1, Lipotes vexillifer; XP_007448903.1, Sus scrofa; XP_005667267.1. Reference sequences of the ZNF408 orthologues: Homo sapiens; NP_079017.1, Macaca mulatta; XP_001111210.1, Felis catus; XP_003993266.1, Canis lupus familiaris; XP_003639743.3, Equus caballus; XP_001490832.1, Pantroglodytes; JAA35243.1 Bos taurus; NP_001180086.1, Callithrix jacchus; XP_009006283.1, Condylura cristata; XP_004683622.1, Sus scrofa; XP_013849819.1, Elephantulus edwardii; XP_006896777.1

Three heterozygous missense changes (p.Pro44Ser, p.Met232Val and p. Glu715Asp) identified in *ZNF408,* were observed in one proband each. Of the total nine missense changes identified in the present study, except p.Pro44Ser and p.Met232Val changes of *ZNF408,* the remaining were predicted as deleterious by at least two computational prediction tools used for *in silico* analysis. Furthermore, the wild type amino acids located at these missense changes were highly conserved across different species (Fig. 2). Thus, these missense changes were categorized as potentially pathogenic mutations, while the p.Pro44Ser and p.Met232Val changes of *ZNF408* were variations of unknown significance.

## Discussion

In the present study, several novel mutations (missense, non-stop and insertion) were detected in the coding regions of *FZD4, TSPAN12* and *ZNF408* genes among the unrelated FEVR probands. None of these nucleotide changes were observed in 115 ethnically matched controls and were not present in the dbSNP [44], ESP5400 [45], NIEHS95 [46] and ExAC [47] databases. The probands harboring the mutations in these genes exhibited severe disease phenotype with neovascularization, falciform retinal folds, exudative or tractional retinal detachments and severe visual impairment. Some of these mutations were also observed in the family members of the probands who exhibited a highly variable phenotype based on indirect ophthalmoscopic examination. The phenotype of the family members ranged from normal retinal vascularization or very mild avascularization to neovascularization, partial and/or total retinal detachments with or without exudation. Since, some of the asymptomatic family members with the identified mutations were unavailable for fluorescein fundus angiography, we could not rule out the possibility of missing very mild retinal vascular changes. The patients identified with *FZD4* or *TSPAN12* or *ZNF408* mutations exhibited classical clinical features of FEVR, but failed to exhibit any genotype-phenotype correlation.

### Mutations detected in the *FZD4* gene

**Fig. 3:**
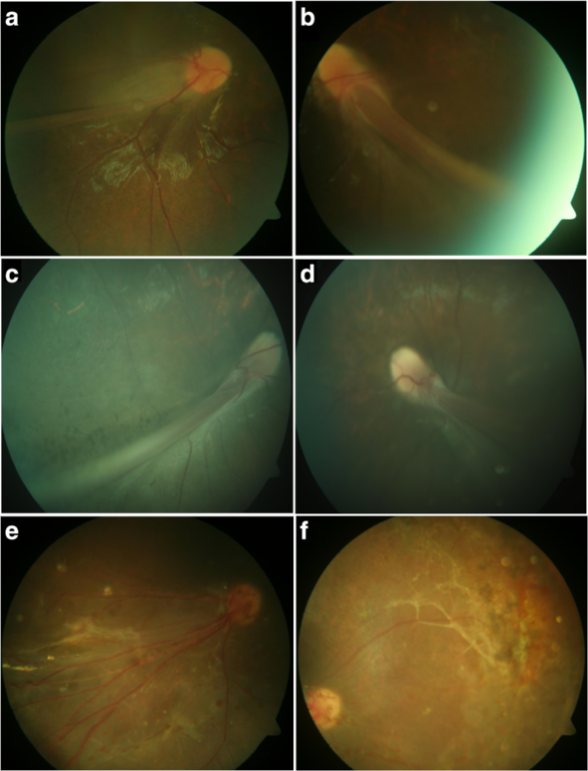
Fundus photographs of the FEVR patients with novel nucleotide changes identified in the *FZD4* gene. **a**&**b** Patient ID: family 1-II:1 (c.341 T > G); Right and left eyes of proband of FEVR family 1 shows straightening of blood vessels and macular dragging toward inferotemporal area of the retina. **c**&**d** Patient ID: family 2- II:1 (c.1395_1396insT); Right and left eyes of proband of FEVR family 2 shows macular dragging toward inferotemporal retina. **e**&**f** Patient ID: family 3- II:3; Right and left eyes of proband of FEVR family 3 shows straightening of blood vessels, exudation and vitreoretinal traction

A total of 58 different mutations have been reported in *FZD4* in FEVR patients, including 30 missense mutations, 10 deletions, 9 nonsense mutations and two singe bp insertions [1, 2, 7, 8, 14–20, 42, 51–55]. A heterozygous novel c.1395_1396insT (p.Arg466Serfs*6) change resulting in a frame shift at codon 466 leading to a premature termination codon, was observed in a familial case of FEVR (Fig. 1; family 2; II:1) that segregated in all the affected family members. The proband and her female siblings (II:1 and II:3) with this frameshift mutation developed bilateral vitreo retinal traction and total retinal detachment at their infancy while their mother (I:2) who was a carrier for this mutation, had avascularized peripheral retina (Fig. 3). This is the third insertion mutation to be observed in the *FZD4* gene so far.

A novel non-stop change (c.1613A > C; p.*538Serext*2) along with a previously reported 4 base pair deletion (c.1282-1285delGACA; p.Asp428Serfs*2) [1, 2, 15, 16] was observed in a familial case in a compound heterozygous condition. This change resulted in disruption of proper termination and addition of two extra amino acids to the wild type protein. A severe disease phenotype with bilateral total retinal detachments was observed in the proband (II:3) and her female sibling (II:1) that might have resulted from the synergistic effect of both the mutated alleles. The father (I:1) and mother (I:2) of these siblings were carriers for the mutated alleles c.1613A > C and c.1282-1285delGACA, respectively The parents of these siblings harboring either of the mutated alleles were normal or exhibited avascularized peripheral retina. The non-stop mutation has been identified for the first time in *FZD4* gene and predicted to result in addition of two extra amino acids to the C-terminal cytoplasmic domain of FZD4 protein. Heterozygous compound mutations have previously been reported in *FZD4* gene and could explain the inter and intra familial variability of disease phenotype [18, 20]. The c.1282-1285delGACA change led to an open reading frame alteration at codon 428 and formation of a new stop codon at position 430.

The third frameshift heterozygous alteration c.1286-1290delAGTTA (p.Lys429Argfs*28) was observed in two unrelated probands in families 4 and 5 (Fig. 1; family 4; II:2 and family 5; II:1). Both the probands developed total retinal detachment in one eye during early childhood and the affected eyes developed subtotal retinal detachment excluding the fovea. The mother (I:2) of the proband 5 who was a carrier for this deletion exhibited peripheral retinal avascularization while the proband’s father (I:1) despite having a single copy of the mutant allele did not exhibit any retinal vascular abnormality upon indirect ophthalmoscopy examination. This deletion led to frameshift at codon 429 along with the formation of a premature stop codon.

All the three frameshift mutations (p.Arg466Serfs*6, p.Asp428Serfs*2 and p.Lys429Argfs*28) were predicted to result in the formation of a truncated protein with a loss of 13 to 20 % of the wild type FZD4 protein. Due to the presence of only two exons in *FZD4*, the frameshift mutations observed in the second exon may result in the formation of truncated protein rather than nonsense mediated mRNA decay [56]. The resulting truncated proteins affected the conserved KTXXXW domain and PDZ binding motif, essential for interaction with downstream regulator proteins. In the absence of these motifs, FZD4 would likely fail to induce the signaling pathway and consequently lead to haploinsufficiency. Alternatively, the truncated protein may oligomerize with wild type protein and become trapped in the endoplasmic reticulum as reported in the p.Lys501fs*533 mutation producing a dominant negative effect [57, 58].

Three missense mutations p.Met105Val, p.Ile114Ser and p.Met157Thr identified in the present study in *FZD4* were located in the extracellular cysteine rich domain (CRD) of the gene. The wild type amino acid residues located at the 105, 114 and 157 codon positions were experimentally found to be crucial for ligand binding during Norrin signal transduction [30, 40, 41]. A novel heterozygous mutation, c.341 T > G (p.Ile114Ser) was identified in a female proband (II: 1) of family 1 (Fig. 1). The proband was noticed to have bilateral nystagmus with exudative retinal detachment including her macula at 8 months of age. The mother of the proband (I: 2) also harbored this change but was found to be clinically normal under indirect ophthalmoscopic examination (Fig. 3). Although, the p.Ile114Ser was identified in the present study, Robitaille et al., [53] reported a p.Ile114Thr mutation in a familial FEVR case with bilateral retinal folds at 2 months of age. The isoleucine residue at 114 codon position of *FZD4* was highly conserved across multiple vertebrate species (Fig. 2). Replacement of hydrophobic isoleucine with a hydrophilic serine residue at codon 114 was predicted to have damaging effect on protein function by three algorithms in *insilico* analysis (Table 1). These findings provide evidence for the possible involvement of p.Ile114Ser mutation in disease pathogenesis.

Both the p.Met105Val and p.Met157Thr mutations have been previously identified in the Caucasian and Asian populations [1, 2, 16, 18, 20]. In the present study, we have identified the replacement of methionine by threonine at 157 codon position in a sporadic case (Fig. 1; family 6; I:2) with total retinal detachment in both eyes of the proband. This patient was examined at the age of 35 years with roving movements in the right eye and bilateral ectopic macula towards the temporal side. Her two sons (II:3 and II:4) were also examined and found to be clinically normal and did not harbor the observed change. In addition to this mutation, a missense change (c.694A > G; p.Met232Val) in *ZNF408* of unknown significance was also detected in the proband and one of her son without any observable retinal non perfusion, indicating that the p.Met157Thr was mainly responsible for the disease phenotype in this family. The methionine at 157 codon position precedes a cysteine (codon 158), which is involved in the formation of a disulfide bond crucial for structural maintenance of CRD [39]. Based on this information, we speculate that the altered solvent interactions of hydrophilic threonine instead of hydrophobic methionine in M157T mutation may interfere with the disulfide bridge formation by following cysteine. Thus, the structural alteration caused by this mutation may interfere with the ligand interaction or *FZD4* homodimer formation and could result in the pathogenicity.

The recurrent heterozygous mutation c.313A > G (p.Met105Val) was observed in 3 different sporadic FEVR probands (Fig. 1; families 7, 8 and 9) with advanced stages of FEVR. All the probands having this mutation developed falciform folds and tractional retinal detachment either in one or both eyes within a few months of their birth. Among the three families, two of the mothers (Families 7 and 9; I: 2) and a sibling of the proband 9 (II:1) failed to show severe retinal abnormalities despite of harboring a single copy of mutant allele. However, the mother of the proband 8 (I:2) showed peripheral retinal avascularization.

Notably, like the M157T, the M105V mutations also affected a cysteine residue at codon 106 that plays a crucial role in CRD structure formation via disulfide bridge formation with another cysteine at codon 45. Replacement of the highly conserved methionine at codon 105 with a valine residue led to severe impairment of *FZD4* signaling (Fig. 2) [30]. This information strongly suggested the pathogenic nature of this missense mutation.

### Mutations detected in the *TSPAN12* gene

**Fig. 4:**
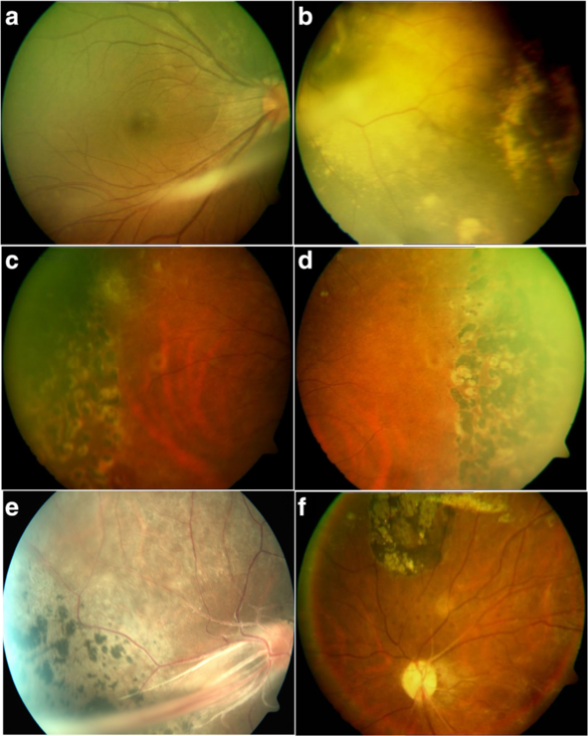
Fundus photographs of the FEVR patients with novel nucleotide changes identified in the *TSPAN12* gene. **a**&**b** Patient ID: family 14-III:2 (c.479G > A); **a** right eye of the proband of FEVR family 14 shows slightly dragged macula. **b** left eye of the proband of FEVR family 14 shows avascular peripheral retina and exudation. **c**&**d** Patient ID: family 14-III:4 (c.479G > A) right and left eye of a family member of FEVR family 14 shows avascular peripheral retina and scars of laser photocoagulation treatment. **e**&**f** Patient ID: family 15-IV:2 (c.334G > A) right and left eyes of proband of FEVR family 15 shows straightening of blood vessels, retinal pigmentation, dragged macula and exudation

So far, 27 different mutations in *TSPAN12* have been reported in FEVR patients [1, 2, 4, 5, 22–25, 59]. These mutations included missense, nonsense, small base pair deletions, insertions and splice site mutations. A novel, heterozygous c.125 T > C (p.Val42Ala) missense mutation was identified in a female proband (IV: 2) of a consanguineous FEVR family 10 (Fig. 1). The proband developed bilateral nystagmus along with attached retrolental membranes to the posterior capsule and funnel shaped retinal folds at 4 months of age. This mutation was also observed in the maternal uncle (III: 3) and aunt (III: 6) of the proband who was reported to be blind since birth. However, both the parents of the proband harboring this heterozygous change were clinically normal. Another novel missense mutation c.479G > A (p.Cys160Tyr) was identified in probands of four unrelated FEVR families (Fig. 1; families 11 to 14). In two probands (IV: 2) of consanguineous FEVR families (11 and 12), this mutation was found in a homozygous condition and showed autosomal recessive inheritance. The probands of the other two FEVR families (13 and 14) were heterozygous for this change. The two probands having homozygous mutation exhibited total retinal detachments with a funnel shaped detached retinas within a few months after birth. The probands with heterozygous mutation progressively developed exudative or tractional retinal detachments with fibrovascular proliferations during the first or second decades of their life (Fig. 4). The other members of these families harboring this mutation showed variable clinical expressivity that ranged from very mild peripheral retinal non perfusion to exudation, falciform retinal folds and total retinal detachments. The three prediction tools used for insilico analysis indicated the damaging effect of this non-conservative substitution on protein function.

The third missense mutation c.334G > A (p.Val112Ile) was identified in homozygous condition in a male proband of FEVR family 15. This patient was first examined in our hospital at 24 years of age and found to have total retinal detachment with falciform retinal folds in both eyes with bilateral nystagmus at the initial examination (Fig. 4). The mother and a male sibling of the proband, who also harbored this mutation in heterozygous state, were asymptomatic with mild pigmentary changes in the retina. This change was previously reported in heterozygous condition as a rare variant by Exome Aggregation Consortium with the frequency of minor allele to be 0.0001 [47].

Another novel missense change, p.Cys160Tyr was prevalent (3.6 % of the FEVR probands) in the present study. This mutation was observed in the probands and their affected family members of four unrelated FEVR families (Fig. 1; families 11 to 14). Two severely affected probands of the FEVR families (11 and 12) had homozygous p.Cys160Tyr mutation while the remaining probands of two FEVR families (13 and 14) and their affected relatives were heterozygous. Compared to probands with the homozygous mutations, the patients with the heterozygous allele showed a slower progression of the disease or relatively less severe ocular phenotype, suggesting the recessive inheritance pattern of the disease and effect of allelic dosage on disease severity. The previously described FEVR patients with homozygous mutations in the candidate genes had severely reduced protein expression that was shown to be responsible for severity and rapid progression of the disease [21, 23, 60].

The p.Val42Ala mutation was located in the small extracellular loop while, p.Val112Ile and p.Cys160Tyr mutations were located in the large extracellular loop of the protein. The wild type cysteine as seen for p.Cys160Tyr mutation, is located at the second position in the highly conserved CCG signature motif of extracellular loop 2 (ECL2) of the TSPAN12 protein (Fig. 2). This cysteine residue was predicted to be involved in the formation of a disulfide bridge with another cysteine residue located at the 181 position of TSPAN12. The ECL2 was suggested to be involved in protein-protein interactions with targeted proteins in the cell membrane [61]. Therefore, the disrupted structure of ECL2 due to p.Cys160Tyr mutation could probably interfere in TSPAN12 interaction with FZD4 protein, thereby affecting the Norrin-FZD4 signaling.

### Mutations detected in the *ZNF408* gene

**Fig. 5:**
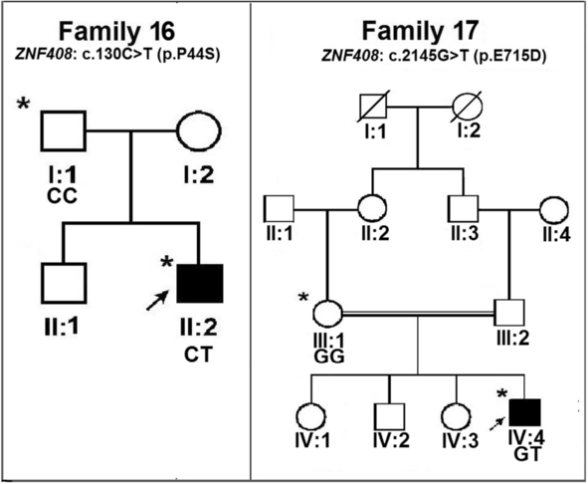
Pedigrees of the FEVR families with nucleotide changes in *ZNF408* gene. Completely shaded symbols represent severe stages of the disease. Open symbols represent unaffected individuals. Asterisk (*) over the pedigree symbols represent the individuals screened for nucleotide changes in *ZNF408* gene. - indicates the presence of deletion/insertion and + indicates the wild type allele. The identified nucleotide change is represented above each pedigree

A novel, heterozygous, c.130C > T (p.Pro44Ser) change was observed in a male proband (II: 2) of FEVR family 16 (Fig. 5). The proband had bilateral total retinal detachments with retrolental membranes at 2 months of age. The father of the proband (I: 2) was found to be normal both phenotypically and genotypically while the mother (I: 1) remained unavailable for genetic analysis. All the three algorithims predicted it to be of benign nature. The second novel heterozygous c.2145G > T (p.Glu715Asp) change was observed in a sporadic proband of FEVR family 17. The proband exhibited total retinal detachment in the right eye and subtotal retinal detachment including macula in the left eye at one year of age. The mother of the proband (I: 2) was found to be clinically normal without this variation, while the father (I: 1) was unavailable for genetic analysis. A previously reported T617N change located in tenth zinc finger domain of the protein was also observed in a family, which could potentially interfere with DNA interaction. However, the presence of this change in the unaffected mother of the proband and in a control individual is suggestive for a less or non pathogenic nature of this variation. In family 6, the proband had M232V missense change in the *ZNF408* gene and also a heterozygous M157T mutation in *FZD4* gene. The heterozygous M232V change was also observed in one of the two unaffected sons of the FEVR proband (Fig. 5) however, but they lacked the M157T mutation of *FZD4.* The data thus suggested that M157T mutation was mainly responsible for the ocular phenotype in the proband. Further structural and functional studies are required to understand the effect of these missense changes on ZNF408 protein and its downstream interactions.

Of the three novel non-synonymous variations observed in the recently identified *ZNF408* gene, the p.Pro44Ser and p.Met232Val changes were predicted to be harmless by the three algorithms used in *in silico* analysis and were considered as variations of unknown significance, while an evolutionarily non-conservative substitution p.Glu715Asp was predicted to be pathogenic (Fig. 2). None of these changes were seen in the controls (Table 1).

Interestingly, none of the variations in *ZNF408* gene in our Indian cohort were observed in the Caucasian and Asian FEVR patients indicating the allelic heterogeneity of this condition. In summary, the present study provides an independent validation of the involvement of *ZNF408* in FEVR from an ethnically diverse population from India. This also expands the mutation spectrum of *ZNF408* in the pathogenesis of FEVR that would need to be further supplemented with diverse data from other populations.

## Conclusion

In summary, the mutation frequencies of the 3 candidate genes among the Indian FEVR patients were comparable to previous reports in *FZD4* (3.5–40 %) [1, 2, 7, 8, 14–20, 42], and *TSPAN12* (3.5–10 %) [1, 2, 4, 5, 22–25] genes in different populations. In previous reports the frequency of FEVR patients with *ZNF408* mutations varied from 0 to 1.8 % in different populations [1–3]. The frequency of patients with *FZD4* mutations in our cohort was relatively higher when compared to a previous study by Nallathambi et al., in another cohort of Indian FEVR patients [42]. This indicates the broad spectrum of *FZD4* mutations in FEVR pathogenesis among the Indian patients. Other than the common ocular features of FEVR, we did not recognize any distinguishable features specific to the cases harboring *FZD4, TSPAN12* and *ZNF408* mutations in these patients for undertaking genotype-phenotype correlation. The novel mutations identified in the present study further broadened the mutation spectrum in FEVR patients, which would facilitate better genetic counseling and diagnostics to treat these patients before the development of severe visual complications.

## Ethics (and consent to participate)

The study was approved by the Institutional Review Board (IRB) of L.V. Prasad Eye Institute, Hyderabad, (Ref no LEC06104) India, and adhered to tenets of the Declaration of Helsinki. A written informed consent for research purposes was obtained from each subject prior to their participation in the study. In case of minors, the consent for participation in the study was obtained from either of the parents.

## Consent to publish

Not applicable as the study does not identify the subjects and no personal information identifying the subject included in the manuscript.

## Availability of data and materials

The data pertaining the study would be available from the corresponding author and principal investigator of the study upon request for any research use.

## Abbreviations

CRD: cysteine rich domain; DNA: Deoxyribose Nucleic Acid; ECL: extra cellular loops; ESP5400: Exome Sequencing Project 5400; ExAC: Exome aggregation consortium; FEVR: Familial Exudative Retinopathy; FZD4: Frizzled 4; IRB: Institutional Review Board; KIF11: Kinesin family member; LRP5: Low density lipoprotein receptor like protein 5; NDP: Norrie disease pseudoglioma; PCR: Polymerase Chain reaction; PolyPhen-2: Polymorphism Phenotyping Version 2; SIFT: Sorting Intolerant from Tolerant; TCF/LEF: T-cell factor/lymphoid enhancer factor; TSPAN12: Tetraspanin-12; ZNF408: Zinc finger prortein-408.

## Additional file

Additional file 1: Primer and Amplification Details for the Three Candidate genes. (DOCX 17 KB)
