# Supplementary material for: Mutation spectrum of the FZD-4, TSPAN12 AND ZNF408 genes in Indian FEVR patients
Source: BMC Ophthalmol. 2016 Jun 17;16:90. doi: 10.1186/s12886-016-0236-y (PMC4912735; doi:10.1186/s12886-016-0236-y)
Supplement: Additional file 1: — Primer and Amplification Details for the Three Candidate genes. (DOCX 17 KB) [file 12886_2016_236_MOESM1_ESM.docx]

| *FZD4* | Exon1 | 1F-GTGCAAACTGGGGGTGTCTG  1R-TAGCCCGAAGGAGACAGCTC | 460 | 61 | 1.5 |
| --- | --- | --- | --- | --- | --- |
|  | Exon 2 | 2.1F-TCAACTCAGCTTTGTGGGAGC  2.1R-GCGGCTGTATAAGCCAGCAT | 436 | 61 | 1.5 |
|  | Exon 2 | 2.2F-CAGGTGATGAAGAGGTGCCC  2.2R-TCCTTTCCCGGCCTACAGTC | 354 | 61 | 1.5 |
|  | Exon 2 | 2.3F-GTTTTCCTACCCTGAGCGCC  2.3R-CGGTGAGGGCATCGAGATTT | 425 | 63 | 1.5 |
|  | Exon 2 | 2.4F-CATCCCCGCAGTGAAAACCA  2.4R-CATGCCTGAAGTGATGCCCA | 421 | 61 | 1.5 |
|  | Exon 2 | 2.5F-GCAACGTGTGTGATTGCCTG  2.5R-TTTTTGATGCTGGGGTCGGG | 440 | 61 | 1.5 |
| ***TSPAN12*** | Exon 2 | 2F-ATGTCCCGTGTTCTCTCTCC  2R-CCAGGGGTGGATTTCTTTGT | 382 | 55 | 1.5 |
|  | Exon 3 | 3F-CAAGATGCAGCAAATGGTAA  3R-TCCAAAAGATCAAGGAAGAGC | 306 | 48 | 2 |
|  | Exon 4 | 4F-TGAGGCATCATGATTGAAAG  4R-CACTGCTCCCTAATCTTGTGA | 341 | 52 | 1.5 |
|  | Exon 5 | 5F-AGGGGCTTCATGAAAACTTG  5R-GCGGAGTGAAAATGAACTAACA | 285 | 57 | 1.5 |
|  | Exon 6 | 6F-GACATTCCGAGTATGCGTGT  6R-GCAGGCCATGAAGTTACCTA | 392 | 60 | 1.5 |
|  | Exon 7 | 7F-TGTGGTTTCTGAGGCTGACT  7R-TTCTTCTGCTTCTCCCCATA | 330 | 55 | 1.5 |
|  | Exon 8 | 8.1F-GCTTTCCCTGAGAACCACTG  8.1R-AAGCTGTTTGCCATGGATGT | 420 | 52 | 2 |
|  | Exon 8 | 8.2F-GGGGACAGACCAAATGATGT  8.2R-TGTCCAGGTGGTGACTTATGA | 341 | 51 | 1.5 |
|  | Exon 8 | 8.3F-CTTGTTTTACTGGACTTGTGAA  8.3R-ATCAGAAGAATAGATCGCTGAG | 428 | 52 | 1.5 |
|  | Exon 8 | 8.4F-TGGAGCCATAGTAAAGGTTGAT  8.4R-TGTGTAATATAAGCCCAGGACA | 419 | 55 | 1.5 |
|  | Exon 8 | 8.5F-ATTTGTCCTGTATAGCATCATT  8.5R-TGATTCTCACAAGCATTTTTC | 386 | 50 | 1.5 |
|  | Exon 8 | 8.6F-GCTTATCTTTGCCTTCTCCAAA  8.6R-GTGGCATAAGTGCTTGTAATGT | 3654 | 55 | 1.5 |

**Supplementary Table 1: Primer and Amplification Details for the Three Candidate genes**

| ***ZNF408*** | Exon 1 | 1F- TATCCCCAACCATTTCCGCC  1R- CTGAGGAGAAAGCTGACCACA | 532 | 60 | 1.5 |
| --- | --- | --- | --- | --- | --- |
|  | Exon 2 | 2F- CGGTTTCCTCCCACACTTTTC  2R-GCTGTCTAGCAATGTCCAAACC | 423 | 50 | 1.5 |
|  | Exon 3 | 3F- GGCTTCTAACCTTCCAGGAGT  3R- CCAAGCAGTCTGGGTCCTAA | 333 | 58 | 1.5 |
|  | Exon 4 | 4F-GAATGTTCCAAAGGCACGGC  4R-AATCAAGCCCCACCCTCCTA | 556 | 60 | 1.5 |
|  | Exon 5 | 5.1F-CATTGCTCCCTCTAAGGCTCA  5.1R-TGCTTCTTTAGGTGGCACAGC | 578 | 56 | 1.5 |
|  | Exon 5 | 5.2F-AGTCTGGCTTCCCTACACTCT  5.2R-CAAATGCCCACGCAGGTTG | 560 | 56 | 1.5 |
|  | Exon 5 | 5.3F-GCTCCATACAGGAGAAAAGCCT  5.3R-TGACCTCAACAACATCCCTGG | 566 | 56 | 1.5 |
|  | Exon 5 | 5.4F-GTGCCTTCTGCTGCTTCTG  5.4R-AGCTCTACCGGATGTCAATTCAA | 469 | 59 | 1.5 |

Primer sequences represented by * were taken from a published report by Meindl *et al*., (1992). Remaining primer sequences were designed by using primer-blast (http://www.ncbi.nlm.nih.gov/tools/primer-blast
